# Research Status and Development Trend of Lower-Limb Squat-Assistant Wearable Devices

**DOI:** 10.3390/biomimetics10050258

**Published:** 2025-04-22

**Authors:** Lin Li, Zehan Chen, Rong Hong, Yanping Qu, Xinqin Gao, Xupeng Wang

**Affiliations:** 1 School of Mechanical and Precision Instrument Engineering, Xi’an University of Technology, Xi’an 710048, China; lilin15158@xaut.edu.cn (L.L.); quyanping@xpu.edu.cn (Y.Q.); gaoxinqin@xaut.edu.cn (X.G.); 2 Department of Industrial Design, Xi’an University of Technology, Xi’an 710054, China; zehan922@163.com (Z.C.); 13759617001@163.com (R.H.)

**Keywords:** squat assist, exoskeleton, motion perception, experimental evaluation, summarize

## Abstract

The accelerating population aging and increasing demand for higher work efficiency have made the research and the application of lower-limb assistive exoskeletons a primary focus in recent years. This paper reviews the research progress of lower-limb squat assistive wearable devices, with a focus on classification methods, research outcomes, and products from both domestic and international markets. It also analyzes the key technologies involved in their development, such as mechanical mechanisms, control strategies, motion sensing, and effectiveness validation. From an industrial design perspective, the paper also explores the future prospects of lower-limb squat assistive wearable devices in four key areas: multi-signal sensing, intelligent control, human–machine collaboration, and experimental validation. Finally, the paper discusses future development trends in this field.

## 1. Introduction

In specific work environments, professionals are often required to maintain standing or squatting postures or alternate between them for extended periods to perform their tasks. These occupational habits can lead to muscle injuries, commonly known as Work-related Musculoskeletal Disorders (WMSDs) [1]. WMSDs result from long-term cumulative damage to the body’s structures, including muscles, tendons, ligaments, cartilage, and nerves. Between 1990 and 2017, work-related musculoskeletal injuries accounted for 38.4% of health life years lost due to lower back diseases [2]. These health issues, resulting from occupational habits, harm both affected professionals and socioeconomic development [3].

Lower-limb wearable devices are considered effective interventions for reducing WMSD risk factors [4,5]. Jobs like frame cutting, parts handling, and assembly welding often require workers to squat or maintain squatting postures. In [6], the authors suggested using exoskeletons to reduce physical load during standing and squatting. Exoskeletons consist of external mechanical structures attached to the body, providing assistance and enhancing the wearer’s muscle strength. Research in [7] explored the potential of seatless chairs in alleviating musculoskeletal issues among surgeons during surgeries. Another study [8] examined the impact of passive lower-limb exoskeletons on physical load, upper body posture, posture control, and discomfort during simulated industrial tasks.

Recent reviews of lower-limb wearable devices have primarily focused on general postural support systems or industrial exoskeletons. For example, reference [9] provided a comprehensive overview of posture-assistive devices but did not explicitly identify squat-assist exoskeletons as a separate category. Similarly, reference [10] conducted a systematic review of lower-limb industrial exoskeletons, focusing on evaluation methodologies, performance, and safety aspects. However, it lacked an in-depth analysis of squat-assist technologies and their specific challenges. Moreover, existing reviews often emphasize mechanical structures or control strategies, while neglecting critical aspects such as multimodal sensing, intelligent control, human–machine interaction, and experimental validation. These factors are essential for improving user experience and ensuring safety, particularly in industrial and rehabilitative applications of squat-assist exoskeletons.

In contrast to previous studies, this review offers a focused and comprehensive analysis of wearable devices designed to support lower-limb squatting. These technologies are systematically classified according to their primary approaches to squatting assistance. Recent advancements are analyzed from four key perspectives: mechanical structure, control strategies, motion sensing, and performance evaluation. From an industrial design perspective, this review also outlines future directions in four emerging areas: multimodal sensing, intelligent control, human–machine collaboration, and experimental validation. By bridging engineering technologies with industrial design, this work provides insights into the development trends of squat-assist lower-limb exoskeletons. By critically reviewing current achievements, unresolved challenges, and future research directions, this study aims to guide further research and support the practical application of these wearable systems in industrial and healthcare settings.

This study employs a systematic literature review to comprehensively analyze the current status and development trends of lower-limb squat-assist devices. The literature were retrieved from Scopus, Web of Science, PubMed, and CNKI, covering publications from 2010 to 2024. The search strategy used multiple keywords, including “lower-limb exoskeleton”, “squat assist”, and “wearable devices”. Boolean logic was applied to refine the search process.

Clear inclusion and exclusion criteria were applied during the literature selection process. Inclusion criteria included journal articles, conference papers, and patents related to lower-limb assistive devices, with emphasis on experimental studies, design innovations, and practical applications published in the past five years. Only publications in English or Chinese were included. Exclusion criteria involved unrelated studies, redundant publications, or outdated content (any literature before 2010 was excluded unless it held significant representative value). In total, 37 articles were selected for review and analysis.

## 2. Lower-Limb Wearable Exoskeleton Seat

**Figure 1:**
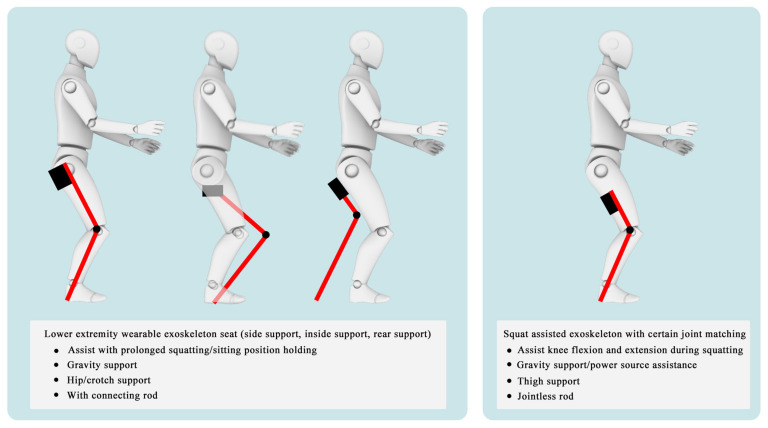
The lower extremity wearable exoskeleton seat and squat-assisted exoskeleton with certain joint matching. Red parts indicate the structural support components of the exoskeleton, while black parts represent the binding areas between the device and the human body.

Lower-limb squat assistive devices must perform two essential functions: providing weight support during dynamic transitions (from standing to squatting and vice versa) and locking the device in a static position [11]. Lower-limb assistive devices can be categorized into wearable exoskeleton seats and squat-assist exoskeletons with joint matching, based on how they address these needs [10]. The lower-limb wearable exoskeleton seat allows users to walk freely to a certain extent and sit at will after wearing it. While ensuring some lower-limb mobility, it provides squat support and assistance at specific angles [12]. In comparison to exoskeleton seats, squat-assist exoskeletons with joint matching offer users a larger range of motion. They can perform asymmetric squat movements, such as kneeling on one knee, which the exoskeleton seat cannot facilitate. However, they cannot provide the same level of support during sustained squatting as exoskeleton seats. The key difference between the two is whether a connecting rod links the support poles of the two legs and whether the device includes a hip support component (as shown in Figure 1). Lower-limb wearable exoskeleton seats are further categorized into side-support, rear-support, and inner-side-support seats, based on the positioning of their primary support components relative to the lower limbs.

**Figure 2:**
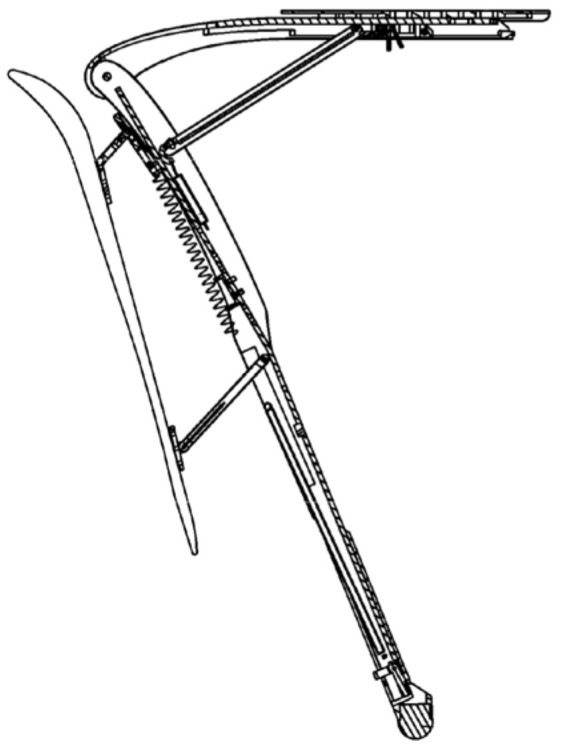
Wearable chair.

Research on lower-limb wearable exoskeleton seats began in 1977 when American designer Darcy Robert Bonnet filed a patent for a “Wearable Chair” [13] (as shown in Figure 2). This device is designed to provide seated support for the user. The core principle of this design is to replicate the human knee joint structure through a hinge mechanism, fixed to the back of the user’s legs and secured with straps.

### 2.1. Side-Support Exoskeleton Seat

The side-support exoskeleton seat positions support rods on the outer sides of the wearer’s legs to provide support [14]. This design distributes weight across the legs, reducing the load on muscles and joints. The side-support structure adapts to small leg movements, offering stable lateral support while maintaining proper alignment with leg joints in the sagittal plane, reducing fatigue or discomfort from improper sitting posture [15]. The focus is on enhancing stability on both sides of the wearer’s legs, particularly aligning the legs with the torso, reducing leg abduction, and maintaining an upright or slightly adducted leg position. A summary of representative side-support exoskeleton seats, including their structure and key features, is provided in Table 1.

The side-support style is relatively uncommon in commercially available lower-limb exoskeleton seats, with LegX as the primary representative. LegX [16], designed by SuitX (Berkeley, CA, USA), supports industrial workers’ squat postures and switches between working modes via an active control system. In dynamic mode, the device uses compression springs to store and release energy, assisting in sitting and standing postures. In static mode, the wearer can lock the device in three positions, transferring their weight to the ground. Experimental evaluations showed that wearing LegX reduced lower-limb muscle activity by 56%. In 2020, SUITX and the University of California, Berkeley [17] evaluated the effectiveness of LegX in simulating work tasks, proving its efficacy in reducing knee pressure and alleviating the burden on the rectus femoris [18].

Currently, side-support exoskeleton seats in laboratory stages primarily use pneumatic or hydraulic power sources, which offer high power-to-weight ratios, fast response times, and good controllability and stability. In 2004, Mitsuda T et al. [19] proposed a wearable seat using pneumatic passive components to reduce physical strain from prolonged standing. In 2016, Wang Z et al. [20] designed a lower-limb assistive exoskeleton hip joint structure for deep squat movements by combining a bias slider–crank mechanism with a hydraulic system, using hydraulic cylinders to drive hip joint flexion and extension. Some researchers have also used brushless DC motors as power sources for squat-assists in side-support exoskeleton seats. In 2016, a research team from Kwangwoon University [9] developed a wearable robotic system incorporating a rotary actuator and a linear spring, based on a four-bar linkage mechanism. The device assists individuals weighing up to 85 kg in maintaining any seated posture, while consuming nearly zero current during walking. In 2019, Sado F et al. [21] designed UMExoLEA, a lower-limb exoskeleton with six degrees of freedom, driven by bidirectional brushless DC motors for sagittal plane movement, and using ground reaction force sensors and joint angle sensors to capture human motion data. In 2024, Ju H et al. [22] designed J-Exo, a lower-limb exoskeleton using telescopic linear actuators as leg actuators and flexible gait-detection insoles to monitor walking cycle phases. Experimental results showed that J-Exo wearers experienced significantly reduced muscle activity during stair climbing and squatting tasks, with work duration increasing by 7.11 times. In 2017, Daines K et al. [23] studied sitting–standing transitions assisted by crutches, integrating crutch support into the squat-assist exoskeleton.

**Table 1:** Side-support exoskeleton seat.

Side-Support Exoskeleton Seat								
Name/ Source	Support and Power Parts	Driver	Locking Mechanism	Quality	Assisting Effect	Graphical Representation	Market Launch	Technology Commercialization Barriers
LegX from SuitX [16]	thigh/ knee	Compression spring	Manual; Three-stage locking	/	Muscle activity decreased by 56%		/	/
Ritsumeikan University, Japan [19]	Buttoc/thigh/knee	Air pump	Auto; Stepless locking	4 kg	Structural support reduces lower-limb loading	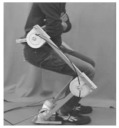	/	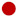 SH (Noise and lack of fall protection devices)
Anhui University of Technology [20]	Hip/ knee	The hydraulic cylinder	/	/	Simulation results indicate hip joint range of up to 135° extension and 30° flexion	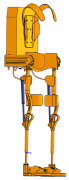	/	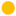 EC 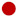 SH (The hydraulic system carries a risk of high-pressure leakage) 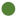 ED (It may lead to motion lag or a sense of impact)
KARE Kwangwoon University [9]	Hip/ knee	Gas Strut	Singularity point of the linkage	6 kg	General muscle activity reduction observed	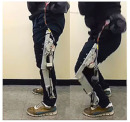	/	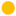 EC (Complex structure)
UMExoLEA, Universities in Malaysia [21]	Hip/ knee	Brushless DC motor	Auto; Stepless locking	/	Muscle activity in medial gastrocnemius and lateral gastrocnemius decreased by 40.8% and 45.3%, respectively	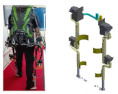	/	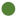 ED (The prototype is relatively heavy)
J-Exo, Harbin Institute of Technology, China [22]	Hip	Electric machine	Auto; Stepless locking	7.7 kg	Average EMG activation of VL, VM, BF, TA, and GAS muscles decreased by 62.02%, 53.36%, 70.04%, 67.50%, and 35.33%, respectively	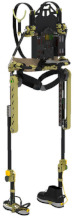	/	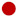 SH (Insufficient force balance) 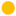 EC (Relies on customized flexible insoles and IMUs)

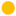
EC—Excessive Cos, 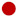
SH—Safety Hazards—SH, 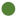
ED—Ergonomic Deficiencies.

### 2.2. Rear-Support Exoskeleton Seat

The rear-support exoskeleton seat places the support rods behind the wearer’s legs to provide support. This design shifts the wearer’s center of gravity backward, transferring the upper body’s weight to the ground via the exoskeleton seat, thus assisting in maintaining an upright posture and reducing pressure on the lower back [24]. The rear-support device provides necessary backward thrust during sitting and standing, making it suitable for users needing additional rearward stability. The rear-support component often integrates power devices such as motors, springs, or pneumatic/hydraulic cylinders, offering timely assistance based on the user’s motion. However, the rear-support exoskeleton seat interferes more with wearer movement than the other two types. During stair climbing, the rear support rods may collide with handrails or steps, especially in narrow staircases. This not only affects safety but also hinders the continuity and efficiency of stair climbing. Furthermore, since the device’s joint axes are not aligned with the wearer’s joint rotation center, discomfort may occur during walking. This is a common issue with rear-support exoskeleton seats [25,26,27]. Table 2 presents an overview of rear-supported exoskeleton seats.

Due to its intuitive use and higher market acceptance, the rear-support exoskeleton seat is the most common type in commercially available lower-limb assistive devices. Noonee’s gravity-supported exoskeleton system, “Chairless Chair” [28], developed by Noonee (Zurich, Switzerland), is considered the first commercially available rear-support exoskeleton seat.is the earliest commercial rear-support exoskeleton seat. The wearer bends the knees to the desired sitting angle and triggers the activation button. The built-in adjustable damper supports the wearer’s weight, transferring it effectively. H-CEX [29], developed by Hyundai Motor Company (Seoul, Republic of Korea), features a four-bar linkage system that passively unfolds with knee flexion, integrating an elastic component to assist in dynamic transitions and provide multi-point sitting assistance. This device reduces lower-body muscle activity by 30.59–84.08% [30]. The Lex [31], introdued by Astride Bionix (Singapore), is a gravity-supported exoskeleton system designed for an ideal sitting experience. It allows the wearer to sit with a straight back, slightly bent thighs, and feet securely planted, unloading the weight on the hips. The “ExoChair” consists of adjustable leg and hip supports, connected by a “corset belt” at the back and “suspenders” over the shoulders [32]. The system is supported by a metal frame and equipped with a pneumatic actuator and an intelligent control system, enabling both standing support and transformation into a seated position. The “OFREES Chair” [33] is a backpack that integrates a foldable exoskeleton made of aluminum, designed for use in various environments. It features a simple structure, weighs approximately 2 kg, supports up to 120 kg, and comes in four sizes.

**Table 2:** Rear-supported exoskeleton seat.

Rear-Support Exoskeleton Seat								
Name/Source	Support and Power Parts	Driver	Locking Mechanism	Quality	Assisting Effect	Graphical Representation	Market Launch	Technology Commercialization Barriers
Chairless Chair, Noonee [28]	buttock	/	Manual; Fixed lock	2 kg	/	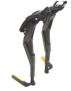	√	/
H-CEX [30]	buttock	/	Fixed multistage locking	1.6 kg	Lower-limb muscle activity reduced by 30.59–84.08%	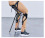	√	/
Lex [31]	buttock	/	Fixed multistage locking	2.2 kg	Effectively relieves gravitational load on the hip joint	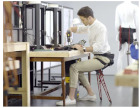	√	/
ExoChair Useful Robotics Russia [32]	buttock	Pneumatic power	/	5 kg	/	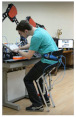	√	/
Ofrees Co., Ltd., Gyeonggi-do, Republic of Korea [33]	Buttock/knee	/	Rotary extreme position	1.9 kg	/	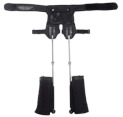	√	/
Flexible wearable Indian Institute of technology, Delhi [34]	/	/	Telescopic bar and lock pin	3 kg	/	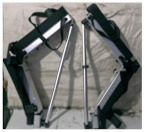	/	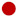 SH (Absence of dynamic stability validation)
HUST-EC, Huazhong University of Science and Technology, China [35]	Buttock/knee	Motor + pneumatic spring	Fixed multistage locking	/	Significantly reduces muscle activation and plantar pressure by 54–67%	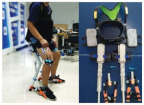	/	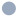 TH 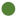 ED (Spring travel-limited posture adjustment)
SW-SiStA, University of Alabama [36]	Thigh/ knee	Pneumatic cylinder	Fixed multistage locking	/	Substantial reduction in knee joint loading; EMG activity of VL, VM, and RF muscles significantly decreased	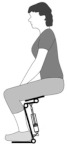	/	/
Chairless chair-based on local component University Tarumanagara [37]	Buttock	/	Slider extreme position	3.7 kg	/	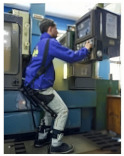	/	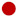 SH (Wearable-device donning predisposes to postural instability)
Beijing University of Aeronautics and Astronautics, China [38]	Buttock/knee	Pneumatic spring	Fixed multistage locking	No more than 5 kg	EMG signals across various muscles decreased by 41–87%, and plantar pressure reduced by 70–80%	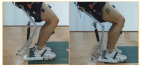	/	/
Shenzhen Institute of Advanced Technology, China [39]	Buttock/hip/ knee	Electric machine	Auto; Stepless locking	/	Simulation analysis shows reduced muscle force demand at the hip and knee joints	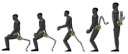	/	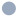 TC 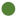 ED
ChairX, Moratuwa University, Sri Lanka [40]	thigh	/	Fixed multistage locking	13.4 kg	Notable reduction in RF and VL muscle activity	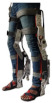	/	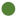 ED (Excessive mass)
E-LEG, Xi’an Jiaotong University, China [41]	buttock	/	Fixed stepless lock	/	Muscle activation reduced during squatting; gait pattern remains largely unaffected during walking	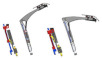	/	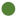 ED Post-donning kinematic constraint of knee joint articulation
Nanjing University of Aeronautics and Astronautics, China [42]	buttock	/	Fixed multistage locking	2 kg	No interference observed between exoskeleton and human limb movement; structural strength meets support requirements	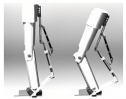	/	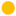 EC 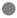 FR (Passive stabilization only, limiting adaptability to complex movements.)
Hunan Normal University, China [43]	buttock	/	Fixed stepless lock	/	Normal coupling maintained between human body and exoskeleton without mechanical interference	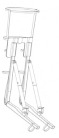	/	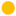 EC 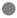 FR

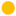
EC—Excessive Cos, 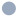
TC—Technological Complexity, 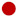
SH—Safety Hazards—SH, 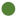
ED—Ergonomic Deficiencies, 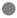
FR—Functional Restriction.

Hydraulic and pneumatic systems typically require considerable space. Rear-support exoskeleton seats utilize the ample space behind the support rods to house these components, supporting high-power system integration and balancing compactness with functionality. In 2016, the team led by Ashutosh Bijalwan developed the “Flexible Wearable” [34], which uses passive control and features a simple structure with easy maintenance. However, it offers limited dynamic response and intelligence. Its innovation lies in dynamic support and adaptability to various postures. An internal hinge mechanism and adjustable locking pins enable smooth transitions between seated and standing positions. The device also adapts to different body types through adjustable linkage lengths and column diameters. In 2019, Han B [35] introduced a wearable exoskeleton chair made of carbon fiber composites and aluminum alloys, integrated with electric motors and pneumatic systems for actuation. Experimental data showed that wearing the device reduced foot pressure by 54–67% and decreased the angle between the upper body and vertical axis by 59–77%. In 2019, Zheng H et al. [36] proposed a novel assistive device, SW-SiStA, using double-acting pneumatic cylinders to drive an inverted crank–slider mechanism. Experiments demonstrated that the peak torque output of SW-SiStA matched the required torque curve, meeting the standing needs of users weighing 47 kg. The chairless chair, developed by Agustinus Purna’s team, features a sliding cushioning system [37]. The frame’s bearings slide along tracks on the thigh support rods. When seated, the support slides to the end of the track and locks. When standing, the bearings are free to move. Weighing only 3.7 kg, the design is especially suited for resource-limited environments. In 2021, Han Y et al. [38] developed a new lower-limb exoskeleton seat mechanism based on a planar four-bar linkage. By controlling the extension of pneumatic springs, the support range was adjustable. Experiments showed that compared to not using the exoskeleton, EMG signals from different muscles decreased by 41–87%, and foot pressure reduced by 70–80%.

When no auxiliary power source is provided for squatting and standing, the functionality of rear-support exoskeleton seats shifts towards offering static squatting support. In this case, the structural design becomes critical, focusing on support stability, adjustable squatting height, and precise locking mechanisms. In 2015, Liao Y et al. [39] introduced a novel adaptive exoskeleton chair utilizing a slider–crank mechanism for dynamic extension and contraction. Simulations confirmed that this design effectively reduced joint load for patients with lower-limb hemiplegia during sitting and standing, decreased energy consumption, and improved motion stability and safety. In 2019, Wijegunawardana I D et al. [40] proposed a robotic exoskeleton chair, ChairX, featuring a locking mechanism at the knee joint and an active connection mechanism behind the legs. Experimental results demonstrated that ChairX maintained stability and effectively reduced musculoskeletal strain. In 2022, Tu Y et al. [41] designed a semi-passive lower-limb exoskeleton for squat assistance, E-LEG, which incorporated a pawl coupled with a push–pull solenoid, enabling the exoskeleton joint to lock at variable heights and adjust squat depth. Experimental results showed that wearing E-LEG reduced muscle activity during deep squats while minimally affecting normal gait. In the same year, Li Chenchen et al. [42] developed a multi-link adjustable lower-limb assistive exoskeleton chair using a six-bar closed-loop mechanism as the core structure. Research indicated that the exoskeleton maintained normal coupling with the human body, provided stable support, and met strength requirements without interfering with movement. In 2023, Kang Huimei et al. [43] developed a non-powered wearable exoskeleton seat with support rods consisting of pneumatic and telescopic rods. During normal gait, the telescopic rods extended and contracted with thigh muscle activity. While squatting, the rods contracted to their shortest length and automatically locked, forming a stable triangular support with the thigh, calf, and pneumatic rods to maintain constant squatting height. During standing or walking, the rods extended based on the squatting height.

### 2.3. Medial-Support Exoskeleton Seats

Medial-support exoskeleton seats assist by positioning support components on the inner side of the wearer’s thighs. This design enhances knee and hip joint stability, especially during adduction or to prevent valgus [44]. In a natural standing or sitting posture, the ground reaction force is directed from both feet toward the center of gravity. Placing the supporting rods along the inner sides of both legs effectively distributes this force [45]. Medial support maintains symmetry in the lower limbs, reducing abnormal stresses on the knees and hips, making it ideal for users requiring enhanced medial stability or undergoing rehabilitation [46]. An integrated summary of internal-support exoskeleton seats is provided in Table 3, illustrating their compact configurations.

The first commercial introduction of medial-support exoskeleton seats was Honda’s Body Weight Support System, developed by Honda Motor Co. (Tokyo, Japan) in 2008. This gravity-support device uses dual DC motors and a transmission mechanism located beneath the seat and behind the wearer. During operation, it generates upward assistive force to alleviate part of the user’s body weight. The exoskeleton’s knee joint rotation center aligns precisely with the wearer’s natural knee joint axis, enhancing comfort. In effectiveness tests, the device showed an 11% reduction in energy consumption and an 18% average decrease in muscle activity [45].

While medial-support exoskeletons are commercially available, many studies remain at the experimental stage. In 2015, Lee K. M. et al. [47] proposed a lower-limb exoskeleton with elastic joints (LEE) to reduce compressive knee loads. This exoskeleton integrates an elastic knee joint with coupling and body weight support functions. In 2018, Wulong H. et al. [48] developed a novel mechanical structure based on a human hinge model and kinematic analysis. They introduced a control method based on plantar pressure and hip contact forces. When using the device, plantar pressure decreased, effectively alleviating stress on the knees and ankles. The measured hip contact force closely matched the preset value, indicating stable and effective support. In 2019, Lovrenovic Z. et al. [49] introduced the assistive exoskeleton concept (WAE), consisting of a cushion and elastic wire unit. Made of aluminum alloy and HDPE, the elastic wire unit connects the hip joint and thigh link unit via a pulley system, generating upward support with mechanical springs. Test results showed the device provided upward support ranging from 9.41% to 26.18% of body weight while standing, and peak forces from 14.02% to 27.52% of body weight during walking.

## 3. Squat-Assist Exoskeletons with Certain Joint Matching

Squat-assist exoskeletons with certain joint matching differ from exoskeleton chairs in that they lack connecting rods between the support rods of both legs, and the support rods may not touch the ground. The key focus of these exoskeletons is fitting the human body’s dimensions and mimicking natural movement patterns. Proper alignment between the exoskeleton’s joint axis and the wearer’s lower-limb joints allows the exoskeleton to closely follow the wearer’s movements, providing smooth, coordinated assistance, minimizing motion interference, and enhancing comfort [50,51]. Table 4 summarizes joint-compatible squat-assist exoskeletons, focusing on biomechanical adaptation and user movement support.

The Archelis gravity-supported exoskeleton system, developed by Yokohama-based Nito (Yokohama, Japan) and Chiba University’s Center for Advanced Medical Engineering (Chiba, Japan), utilizes gravity support and features a highly ergonomic design [52]. It ensures that wearers can maintain suitable postures based on personal habits, fully wrapping around the thighs and calves to distribute body weight through support from the femur and tibia. The exoskeleton knee joint’s rotation center aligns perfectly with the wearer’s natural axis of rotation, resulting in a more comfortable wearing experience.

In 2019, Cui Jiashuo [53] conceptualized the knee joint as a hinge structure, using a motor-driven slider to control the self-locking state of a ratchet through threaded engagement. Experimental results showed that wearing this exoskeleton while maintaining a 90-degree squat reduced plantar pressure by 65.16%, and decreased the forces on the rectus femoris and vastus lateralis muscles by 54.05% and 32.8%, respectively. In 2021, Chen S. et al. [54] designed an electromechanical assist system to provide kneeling assistance for construction workers. The device used QDD (Quasi-Direct Drive) actuators and custom torque sensors, with a low gear ratio transmission to achieve low output inertia. This facilitated low impedance, enabling the system to provide assistance without hindering natural human movement. In the same year, Yan Z. et al. [55] developed and tested a passive lower-limb support exoskeleton. The exoskeleton used torsion springs to store energy generated during squatting and released it to assist the user in standing up. Experimental results showed that using the exoskeleton reduced muscle activity by 44.8–71.5%, plantar pressure by 58.5–64.2%, and increased endurance time from 2.76 min to 13.58 min. In 2022, Zhang Xuan et al. [56] designed a knee-assist orthosis using a self-developed miniature silicone oil spring. The device featured an X-shaped bionic linkage and a gear transmission mechanism, with a flexible transmission structure involving a steel cable and cam mechanism working together. Experiments showed that this device reduced femur–tibia joint force by 24.5%, patella–femoral condyle reaction force by 23.8%, and quadriceps–ligament force by 21.2%. Because human knee joints do not rotate about a fixed axis, joint misalignment is a common issue with assistive exoskeletons. In 2016, Hasegawa Y et al. [57] developed a wearable assistive device with a dual-support structure, distributed around the center of gravity to reduce constraints on hip movement. It uses gas and helical springs to deliver assistive force. To address this, in 2022, Shuangyue Yu et al. [58] designed a bionic knee exoskeleton with a rolling joint mechanism and a high-stiffness flexible cable transmission system. The design aimed to achieve a lightweight construction and high compliance. Simulation results showed that the design could reduce maximum deviation by 49.3% during walking and 71.9% during deep squatting.

Each type entails a unique balance between biomechanical support, user comfort, structural complexity, and practical deploy ability. This paper provides a multidimensional comparative analysis (as shown in Table 5), covering technical strengths, user experience, application contexts, limitations, usability, cost, engineering challenges, and commercialization potential. This structured overview aims to assist researchers and developers in selecting and optimizing appropriate designs based on specific application needs.

## 4. Key Technology Analysis

### 4.1. Kinematic and Dynamic Analysis

The human body has a complex and diverse structure. As a wearable assistive device, the primary task of an exoskeleton is to design a human-centered mechanical structure that ensures no potential harm to the wearer’s body. The human skeletal system provides a solid framework, with fixed bone shapes and relatively limited ranges of motion [59]. Therefore, during the early stages of exoskeleton design, the morphology, positional relationships, and allowable ranges of motion of the bones must be carefully considered to ensure that the exoskeleton does not interfere with normal bone movement. By studying the movement patterns of the human body and clarifying the changes in joint angles of the hip, knee, and ankle during walking or other physical activities, the overall status of lower-limb movements can be directly assessed [60]. Kinematic and dynamic analysis of lower-limb exoskeletons forms the basis for research in structural design, intention recognition, control strategies, and effectiveness verification [61].

When analyzing the positional and directional relationships between the angles of various lower-limb exoskeleton joints and the end-effector components, kinematics can be divided into forward and inverse kinematics. The Denavit–Hartenberg (D–H) parameter method [62] is the most commonly used approach for modeling forward kinematics in lower-limb exoskeletons. Its core is to establish spatial motion equations based on link coordinates, and by using homogeneous transformation matrices, it precisely describes the relationships of objects in motion and transformation spaces. In 2016, Mohd [63] and others simplified the 3-degree-of-freedom lower-limb exoskeleton into a three-bar mechanism using the D–H method for modeling, obtaining kinematic information such as the position and posture of the exoskeleton’s components.

Dynamic analysis of the human lower limbs involves establishing and solving related models to obtain information such as joint forces, joint torques, work, and power. Common dynamic modeling methods include the Newton–Euler method [64], Lagrange method [65], Roberson–Wittenburg method [66], and Kane’s method [67]. For more complex movements, multi-body dynamic simulation software such as Adams (version 2022, MSC Software, Hexagon) and RecurDyn (version V9R5, FunctionBay Inc.) [68,69] can be used for analysis. In 2018, Qi Qiangqiang et al. [70] analyzed the design of a wearable seat mechanism and its kinematics and dynamics, which helped improve understanding of wearable seat mechanism design and related knowledge in the medical research field, ensuring a smooth design process. In 2020, Guo Zhanteng from Xinjiang University [71] performed kinematic analysis of symmetric and asymmetric squat movements of both knees, extracting kinematic features and establishing a mathematical model of squat motion based on the Lagrangian function. This provided parametric support for exoskeleton structural design and theoretical guidance for control of exoskeleton assistance. Existing research methods often overlook the wearer’s muscle biomechanics and the influence of the nervous system on limb movement [72]. Combining musculoskeletal models with finite element methods could enhance model construction accuracy [61].

The results of kinematic and dynamic analysis can also serve as input for equipment design. In squat movements, the knee joint does not have a single, fixed “instantaneous center of rotation” [73]. Knee movement involves relative motion between the femoral condyle (the lower end of the femur) and the tibial plateau (the upper end of the tibia), as well as between the patella and femur [74]. Motion capture technology, electromyography (EMG) signal capture systems, and other methods are usually used to analyze the knee joint’s movement trajectory, angle changes, and biomechanical characteristics, inferring the knee joint’s motion trajectory in the sagittal plane during squatting. Applying this to exoskeleton design can improve motion adaptability, enhance the assistance effect, and increase comfort and safety.

In 2013, Besir Celebi et al. [75] designed the ASSISTON-KNEE, which assists with knee flexion and extension and adapts to knee translation in the sagittal plane. It can automatically adjust the joint axis, achieving ideal alignment between the exoskeleton’s axis and the human knee joint axis, fulfilling ergonomic requirements and ensuring comfort during use. In 2014, Chen Bing [76] and others used a fifth-degree polynomial to fit human knee gait data, analyzing knee joint movement and driving characteristics, and designed a bionic, energy-saving, vibration-damping knee joint actuator. In 2018, Zhu A. et al. [77] designed a passive, lightweight, easy-to-wear weight-support exoskeleton. They used bionic curve modeling and human–machine multi-link design, incorporating a three-position sitting slider mechanism to adapt to various conditions. In 2021, Song Jiyuan et al. [78] established a nonlinear mapping between swing-related joint angle velocities and the joint torque generated after compensating for the human body’s gravitational force, using S-curve mapping and admittance control models. They designed a lower-limb assistive exoskeleton system.

### 4.2. Human Motion Perception

In the use of lower-limb assistive wearable devices, it is crucial for the device to perceive the user’s intentions and take corresponding feedback actions [79]. Instantaneous capture and accurate prediction of human intent are key elements essential for providing effective support and promoting recovery [60]. Currently, apart from using bionic curve simulations of joint motion, active collaborative control methods for lower-limb wearable devices primarily rely on bioelectrical or motion signals for interactive control strategies.

#### 4.2.1. Bioelectrical Signal-Based Interactive Control

Bioelectrical signal-based control, also known as bioelectrical or neural information perception, mainly involves electromyogram (EMG) and electroencephalogram (EEG) signals, as well as signals like brain oxygenation, tongue electrical signals, and eye electrical signals [80]. During voluntary movement, the motor commands are sent by the cerebral cortex, which then transmit as bioelectrical signals through nerve fibers to the muscles. These signals stimulate receptors at synapses, causing action potentials and muscle contractions, resulting in movement [81]. The bioelectrical signals captured from the human body directly reflect movement intentions, enabling the system to realize the “thought-action” motion state [82].

The method of detecting surface EMG signals, using electrodes attached to the skin, is simple and provides relatively accurate signal perception, making it widely applied. Shigeru Inoue and colleagues from Osaka Institute of Technology [83] proposed a method using multivariable regression analysis combined with trunk angle and lower-limb EMG signals to predict the user’s transition time from sitting to standing, enhancing the efficiency of the assistive system. Jin Xinqin and colleagues from Harbin University of Science and Technology [84] conducted research on the impact of squat posture on leg muscle fatigue using surface EMG signals, analyzing signals from the human leg during squat tasks at various angles (0°, 15°, and 25°). Han Yonglin and others [85] proposed a method to capture lower-limb joint motion states in real-time under different movement modes using EMG signals, establishing a mapping relationship between sagittal plane motion and EMG signals. They also optimized the neural network model using the Sparrow Search Algorithm (SSA), enabling more accurate predictions of changes in ankle, knee, and hip joint angles.

However, bioelectrical signal control strategies based on EMG and EEG have certain limitations. Different EMG signals have varying effects on single-joint angles, and the parameters of human skeletal muscles are difficult to measure, making modeling challenging [86]. Therefore, scholars from domestic institutions have proposed models to explore the relationship between EMG signals and joint angle changes. For example, Professor Zhang Xiaodong’s team from Xi’an Jiaotong University [87] developed a Hill muscle force model and joint geometry parameter model, using EMG signals to estimate joint torque. This led to a precise method for perceiving the user’s motion state based on surface EMG signals. Professor Li Wenfeng’s team from Wuhan University of Technology [88] analyzed the impact of different combinations of EMG signals on joint angle estimation, confirming the optimal model degrees of freedom and EMG signal combinations.

#### 4.2.2. Motion Signal-Based Interactive Control

Motion signal-based interactive control refers to using signals generated by human body movement (such as joint angle changes, acceleration, etc.) to facilitate interaction between humans, machines, and the environment [89]. It is mainly divided into position/force control and impedance control.

Position control involves comparing the actual deviation of the motion trajectory or velocity with preset values and controlling the robot to precisely follow the planned movement path, guiding the human limb along the intended path [90]. Force control refers to controlling the force/torque acting on the end-effector or joint actuator in contact with the human body, based on force/torque information captured by sensors [91]. This provides a direct reflection of the user’s subjective motion intention, making it both reliable and stable. Position–force hybrid control combines both position and force/torque deviations as feedback signals to control rehabilitation robots [92]. Impedance control uses errors in position, velocity, and acceleration as feedback signals to control the relationship between the robot’s end-effector motion and the contact force, enabling dynamic control during motion. As such, impedance control is more actively compliant, resulting in more comfortable interaction with the user. Ozer Unluhisarcikli and colleagues [93] developed the ANdROS active knee rehabilitation system, using an impedance controller to apply corrective torque to the wearer based on deviations from the reference knee trajectory. Wei Wei and others [94] introduced a power controller in the design of a hip-assist exoskeleton, combining human joint angular velocity and torque to output adaptive torque according to the wearer’s motion.

Additionally, some researchers have used data collected from exoskeletons (or active orthoses) to accurately estimate and track the wearer’s motion intentions. The approach involves equipping the hip, knee, and ankle joints with force and torque sensors to precisely measure the force and torque exerted by the exoskeleton on the user [95]. The key to realizing this strategy lies in the need for highly sensitive responses to the forces and torques applied to the wearer and accurately calculating the dynamic characteristics of the exoskeleton system. This places stringent requirements on constructing a high-precision exoskeleton model [96]. For example, Ekso Bionics’ Ekso G system monitors joint angle changes in real-time by installing high-precision angle sensors at the knee and hip joints, adjusting the motor output torque to provide appropriate assistance during knee extension based on knee flexion angles [97].

### 4.3. Structural and Mechanism Innovation

The primary purpose of squat-assist wearable devices is to enhance the wearer’s lower-limb strength and assist with squat and stand-up movements. The advantages of four different structural forms of squat-assist wearable devices were discussed in the previous sections. In contrast, the primary focus of device design at the mechanical level is structural optimization to meet the requirements for lightweight, high strength, and compatibility with joint motion characteristics. The mechanical structure design forms the overall framework of the exoskeleton and directly affects its operational efficiency [98]. Ergonomic principles must be considered during the design process to ensure wearer comfort. Size adjustability, support stability, pressure distribution during wear, and joint mobility can be enhanced through structural optimization, component integration, and coordinated positioning. Simulations of the joint range of motion and movement trajectory during squat and stand-up actions should be conducted to ensure smooth and natural device operation. As a wearable assistive device, it must satisfy safety, stability, durability, and lightweight design requirements. The cross four-bar linkage mechanism can further adapt to the changing trajectory of the knee joint’s instantaneous center of rotation [99]. Finite element analysis software can be used for static analysis of the equipment components, and topology optimization can be applied to achieve a lightweight design [100]. During the squat movement assistance process, the device must help the wearer complete three stages: descending into a squat, holding the squat position, and standing up. The motion trends of these three stages differ, and the design of energy-locking and switching mechanisms is crucial for the exoskeleton’s mechanical structure. The self-locking mechanism is critical for maintaining the relative position between the exoskeleton and the wearer’s lower limb. Self-locking mechanisms can be used to lock the knee joint at a specific angle, ensuring the stability of the squat posture [53]. As shown in Table 6, comfort design criteria play a crucial role in user acceptance and long-term usability.

### 4.4. Drive Mechanism Design

In assisting with the squat movement, the selected drive mechanism must accommodate the human body’s weight and the high power required for squat and stand-up motions. In some rapid or high-load scenarios, the maximum torque required to complete the squat and stand-up movements at the knee joint can reach up to 250 nm [101]. Drive mechanisms commonly used in industry for squat-assist wearable devices, similar to lower-limb exoskeleton robots, primarily employ rigid drive methods, including servo motors, hydraulic drives, pneumatic cylinder drives, and pneumatic artificial muscle drives [102]. Among these, motor drive technology is well-established and can achieve high precision. The system enables precise control of output timing, amplitude, and linear variation, enhancing suitability for the power demands of squatting and standing movements [103]. However, its heavy structure and redundant components reduce portability and impose limitations on energy efficiency and operational endurance. Hydraulic drive, due to its larger volume, is often used in exoskeleton seats, providing assistive force to joints via the expansion and contraction of hydraulic cylinders [104]. During the squatting phase, hydraulic cylinders absorb energy and store it in accumulators, releasing it during the standing phase, similar to the principle of linear or pneumatic springs. Motor drive, when combined with the appropriate control system, allows precise control over output time, magnitude, and linear variations, making it more adaptable to the power required for squat and stand-up motions [103]. Both cylinder-driven and hydraulic systems can deliver high instantaneous output force and demonstrate stable performance. However, cylinder-driven systems have slower response times and rely on external air sources, which reduces portability. In contrast, spring-driven systems offer a compact structure and low development and maintenance costs, though they generate limited driving force. They are suitable for applications demanding lightweight design, low cost, and operation in confined spaces. Compared to mechanical springs, pneumatic springs provide greater adjustability and are more widely adopted in commercial applications. Future squat-assist exoskeleton actuators are expected to evolve toward multi-modal integration, lightweight construction, and scenario-specific customization. Table 7 provides a comparison of different drive modes used in lower-limb assistive exoskeletons.

### 4.5. Exoskeleton Effectiveness Validation Methods

Effectiveness validation is the process of evaluating exoskeletons to ensure they can safely assist users in completing squat and stand-up movements without causing harm. Additionally, it is essential to ensure that the device improves squat and stand-up abilities and reduces muscle fatigue. Effectiveness validation can be performed using various methods, which are primarily divided into subjective evaluations, objective experiments, and computer simulations. In [105], a multidisciplinary evaluation method and set of indicators for industrial exoskeleton devices, using multi-modal measurements to gather more effective evaluation data from ergonomics, kinematics, and user experience perspectives was proposed. This approach aims to overcome the limitations of focusing on a single metric and enhances the comprehensiveness of wearable chair usability evaluations.

#### 4.5.1. Numerical Simulation

Computer simulations for lower-limb exoskeleton seat analysis can effectively obtain mechanical and dynamic data for the device, helping assess its feasibility, load-bearing performance, and comfort [106]. Computer simulation analysis requires a three-dimensional model of the device, with finite element analysis performed using simulation software such as ANSYS (version 2022 R2, ANSYS Inc.), ADAMS (version 2022, MSC Software, Hexagon), or SOLIDWORKS (version 2023, Dassault Systèmes) to draw conclusions. SOLIDWORKS software [107] focuses on 3D modeling, enabling quick static simulation results within the design environment, though the solution precision is relatively limited. ADAMS allows for more precise simulation and analysis of the mechanical system’s motion [108], while ANSYS excels in structural and random vibration analysis, providing new methods for reliability analysis of mechanical structures [109].

#### 4.5.2. Objective Experiments

**Figure 3:**
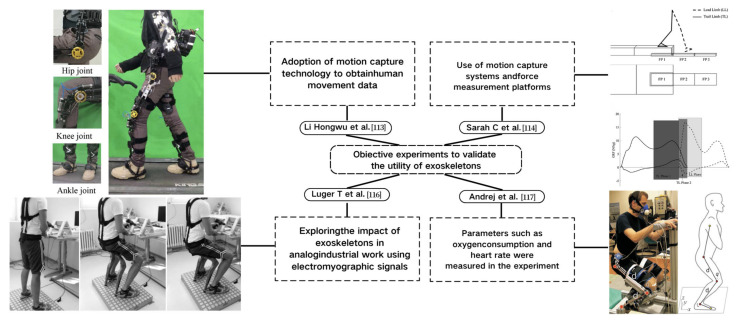
Objective experiment to verify the effectiveness of exoskeleton.

Compared to subjective evaluations, objective experiments involving motion capture, pressure testing, sensor detection, and medical imaging monitoring provide more intuitive and precise validation of exoskeleton effectiveness (as shown in Figure 3). Li, Hongwu et al. [110] utilized markers placed on target objects or human joints to track and compare movement data for users with and without wearing the exoskeleton. Their analysis revealed that the mechanical exoskeleton caused minimal misalignment in the movement pattern relative to the human body. By measuring ground reaction forces, they examined the relationship between foot pressure interactions and lower-limb biomechanics [111]. Similarly, Sarah C et al. [112] employed motion capture systems and force measurement platforms to measure differences in knee joint load during the descent phase, particularly comparing toe and heel contact. From a biomechanics perspective, surface electromyographic (EMG) signals are closely related to joint motion, making them useful in human–machine interaction control. The integration of surface EMG signals for this purpose has been gaining increasing attention from researchers [88].

To further assess the movement intentions of the lower limbs or torso, bioelectrical signals, such as surface electromyography (EMG), can be incorporated into validation experiments. The authors in [113] indicated that the multivariate linear regression accuracy of integrating local EMG signals to estimate oxygen consumption reaches 96%, demonstrating a strong correlation between the electrical signals of active muscles and energy expenditure during movement. Luger T et al. [114] explored the effects of wearing passive lower-limb exoskeletons on body load, upper body posture, posture control, and discomfort during simulated industrial tasks using EMG signals. Medical imaging and cardiopulmonary function testing devices also play a role in exoskeleton effectiveness validation. By measuring metabolic expenditure and heart rate, researchers can compare the energy consumption of performing identical movements before and after wearing the exoskeleton. Andrej et al. [115] studied the impact of a knee-joint assistance exoskeleton on human metabolic costs, finding that the exoskeleton significantly reduced energy consumption during squatting. Liên Wioland et al. [116] reviewed methods and standards for evaluating industrial exoskeletons, identifying limitations such as the focus on short-term effects, the lack of diverse experimental environments, and the predominance of male subjects in current studies.

#### 4.5.3. Subjective Evaluation

Subjective evaluation, in contrast, involves users assessing their experience based on personal feelings and perceptions. Test subjects are asked to wear the exoskeleton according to the experimental instructions and provide feedback about their subjective experience. Typically, a subjective rating scale is used to assess comfort, with participants scoring various subjective indicators. These scores are then statistically analyzed and processed [117]. This subjective measurement of usability and comfort helps to understand how users perceive the device, including its wear resistance, stability, and convenience [118]. Subjective evaluations are valuable for understanding user perceptions and are crucial for assessing the potential of the device for widespread worker use. In tests of CEX exoskeleton effectiveness, researchers found statistically significant results from users’ subjective ratings of the device. A study [119] suggested that exoskeletons should ideally be evaluated during long-term use to reflect real-world daily usage in industrial settings and to account for changes in subjective attitudes and physical adaptation over time. In 2020, Cha J.S. et al. [120] used focus group discussions, simulated surgical tasks, and usability questionnaires to identify potential needs and barriers in using exoskeleton technology to alleviate musculoskeletal (MS) symptoms among operating room staff, finding 17 factors that influence exoskeleton use among surgical teams.

## 5. Outlook

Squatting posture assistive devices, a key component of lower-limb exoskeletons designed to alleviate work-related musculoskeletal disorders (WMSDs), have garnered significant attention from global companies, universities, and research institutes. Several products have already been marketed and have undergone multiple iterations. Exoskeleton research is a multidisciplinary field that encompasses mechanics, materials, bionics, control, electronics, and ergonomics. It requires not only solid theoretical knowledge but also the continuous advancement of related technologies. However, practical applications still face significant challenges, particularly in terms of safety, usability, ergonomic fit, adaptability to real-world environments, and compliance with international standards.

This review classifies current solutions into two main categories: lower-limb wearable exoskeleton seats and joint-compatible squat-assist exoskeletons. A comparative analysis is conducted to evaluate their technological readiness, functional performance, user applicability, and compliance with established safety standards. Exoskeleton seats are relatively mature in terms of commercialization, providing passive support for low-mobility tasks. In contrast, joint-compatible designs offer superior biomechanical alignment but face challenges related to control complexity and ergonomic optimization.

Based on insights from current research and product development, this review identifies five key future research directions and their potential value:

(1) Multimodal Signal Sensing

Multimodal signal sensing combines diverse sensors and processing techniques to monitor and interpret lower-limb movements, enabling more accurate assistance and responsive feedback. Most current systems rely on single-modal sensing approaches, such as inertial measurement units (IMUs) or electromyography (EMG). While effective for basic movement detection, these methods lack redundancy and robustness. Future research should focus on integrating IMUs, EMG, force sensors, and visual feedback systems to enhance signal redundancy and improve motion detection robustness. Effective multimodal fusion requires a clear definition of each signal source’s functional role and robust algorithms to ensure data accuracy and complementarity. Incorporating machine learning algorithms to improve pattern recognition and decision-making is also a promising direction. However, challenges such as signal noise, sensor placement, and data synchronization persist, especially in complex and dynamic work environments. Future work should focus on improving real-time performance, interference resistance, and minimizing discomfort caused by sensor wear. These improvements will support more accurate motion intent recognition, laying a foundation for adaptive and personalized assistance.

(2) Intelligent Control

Intelligent control depends on advanced algorithms and real-time feedback systems, allowing the device to adapt to the user’s specific needs and physical conditions. Traditional control strategies, such as PID and impedance control, provide stability and simplicity but often underperform in complex or dynamic environments. The primary task is to integrate data from multiple sensors and effectively process complex signals to determine the lower-limb movement state. A key component of intelligent control is adaptive control algorithms, which overcome the limitations of single strategies [121] by enabling real-time analysis and automatic adjustment of the device’s response. The integration of adaptive control, deep learning, and reinforcement learning can enhance system responsiveness and improve real-time processing of complex signals. Additionally, improving user intent recognition through multimodal sensing can significantly enhance interaction efficiency between the user and the device. The main challenge is developing scalable and robust algorithms that can adapt to diverse users and dynamic environments. Future research should also focus on optimizing energy use, enhancing computational efficiency, and ensuring long-term reliability. Comparative evaluation of control strategies across varied scenarios will be essential for establishing best practices and improving both safety and energy efficiency in real-world applications.

(3) Human–Machine Collaboration

Human–machine collaboration must begin by ensuring that the assistive device aligns with the biomechanical characteristics of the human lower limb. This requires the device to replicate the natural trajectories and joint motion ranges of squatting and standing, thereby minimizing discomfort and injury risks while ensuring high joint compatibility. The design of human–machine collaboration must prioritize user safety and comfort. This includes adopting lightweight structures, reducing friction, and minimizing pressure or discomfort during wear to enhance user comfort [122]. Incorporating topology optimization or flexible wearable actuation systems can reduce the device’s weight and lessen its impact on user movement patterns and energy expenditure [123]. A comprehensive understanding of user needs is essential, particularly with respect to variations in body size, gender, and occupational requirements. Customizable and modular designs can greatly enhance the device’s adaptability and user satisfaction. Field testing in real-world industrial and rehabilitation environments is crucial for validating the effectiveness of human–machine collaboration. These design strategies have a direct impact on long-term usability and user compliance.

(4) Experimental Validation

The experimental validation of lower-limb squat-assistive devices is a systematic process comprising multiple stages and test types. It includes subjective evaluations, objective measurements, and early-stage stakeholder assessments to ensure that the device addresses key user needs and is suitable for real-world deployment. Currently, most devices lack pre-design validation, which should encompass risk assessment, regulatory compliance checks, performance testing, and user requirement verification. Experimental validation should integrate multiple approaches, combining software simulations, physical measurements, and physiological testing to ensure accurate and comprehensive results. Future research should prioritize early-phase risk assessment, regulatory compliance, performance evaluation, and user requirement validation. Validation should span a variety of use scenarios, such as static squatting, dynamic postural transitions, and diverse industrial tasks, to ensure both reliability and versatility. Moreover, integrating physiological assessments, simulation models, and physical testing will enhance the precision and scope of validation outcomes. Developing standardized evaluation protocols and benchmarking frameworks is also critical for comparative studies. This approach facilitates regulatory approval and builds user trust.

(5) Compliance with International Standards

Ensuring compliance with international standards remains a critical yet often overlooked aspect of current research. Standards such as ISO 13482 [124] (Safety Requirements for Personal Care Robots), OSHA regulations, and CE marking directives provide essential frameworks to ensure safety, reliability, and usability. Future research should establish protocols to ensure standard compliance throughout the development lifecycle, including risk assessments, safety testing, durability evaluations, and ergonomic analyses. Exploring certification pathways for commercial deployment and comparing compliant with non-compliant devices will yield valuable insights for future development. Adherence to these standards improves user safety, product quality, and market credibility. Researchers should actively collaborate with certification and regulatory bodies to ensure design compliance.

Practically, there is an inherent trade-off between functional complexity and industrial deploy ability. Exoskeleton seats offer simpler control and rapid adoption for low-mobility tasks, whereas joint-compatible systems are more suitable for dynamic squatting and rehabilitation, albeit requiring further technical refinement. Future development should prioritize scenario-driven, user-centered design by aligning functional innovation with specific industrial requirements and applicable regulatory standards.

This reflects a trade-off between functional sophistication and real-world applicability. For industrial deployment, systems emphasizing comfort, modularity, and compliance with safety standards are more likely to be adopted quickly. For rehabilitation or specialized applications, intelligent control with higher precision and customization will be essential. These insights should guide future research and development toward scenario-specific, evidence-based design strategies.

In conclusion, the advancement of squat-assist lower-limb exoskeletons requires interdisciplinary collaboration that integrates engineering innovation, ergonomics, control systems, and regulatory compliance. By integrating advances in sensing, control, human–machine interaction, validation, and standardization, future research can deliver robust, adaptable, and safe solutions—enhancing occupational health, improving rehabilitation outcomes, and facilitating broader real-world adoption.

## Figures and Tables

**Table 3:** Internal support exoskeleton seat.

Medial-Support Exoskeleton Seats								
Name/Source	Support and Power Parts	Driver	Locking Mechanism	Quality	Assisting Effect	Graphical Representation	Market Launch	Technology Commercialization Barriers
Body weight Support System, Honda [45]	Buttock/hip/ knee	Dc motor	Auto; Stepless locking	6.5 kg	The device reduced energy consumption by 11% and average muscle activity by 18%	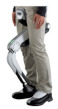	√	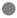 FR 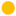 EC (The battery life is limited during extended operational periods)
LEE, Georgia Institute of Technology, USA [47]	Buttock/knee	spring	/	2.357 kg	Effective in alleviating knee joint stress during walking	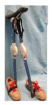	/	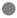 FR (Designed exclusively for level terrain walking)
Peking University, China [48]	buttock	Brushless DC motor	/	/	Experimental results show pressure relief at the knees and ankles	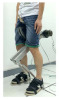	/	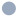 TC (The control system is highly complex)
WAE, University of Ottawa, Canada [49]	Buttock/hip/ knee	Mechanical spring	/	5.68 kg	Provides 9.41–26.18% body weight equivalent upward support in standing, and peak support of 14.02% during walking	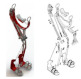	/	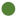 ED (Asymmetric load distribution between left and right legs)

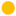
EC—Excessive Cos, 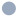
TC—Technological Complexity, 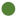
ED—Ergonomic Deficiencies, 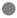
FR—Functional Restriction.

**Table 4:** Squat-assisted exoskeleton with certain joint matching.

Squat-Assist Exoskeletons with Certain Joint Matching								
Name/ Source	Support and Power Parts	Driver	Locking Mechanism	Quality	Assisting Effect	Graphical Representation	Market Launch	Technology Commercialization Barriers
Archelis, Nito [52]	leg	/	Single lock	3.3 kg	Muscle activity significantly reduced	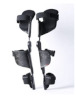	√	/
Huazhong University of Science and Technology, China [53]	knee	/	Auto; Stepless locking	1 kg	Plantar pressure decreased by 65.16%, Rectus femoris and vastus lateralis activity reduced by 54.05% and 32.8%, respectively	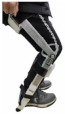	/	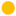 EC 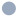 TC (High-Fidelity Dynamic Control)
Rutgers University, USA [54]	Thigh/ knee	Electric machine	/	1.7 kg (unilateral)	Knee extensor/flexor activity reduced by up to 39%, and Knee–ground pressure reduced by up to 15% in the one-leg kneeling posture	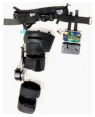	/	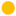 EC (QDD Actuator and Sensor System)
Huazhong University of Science and Technology, China [55]	Loin/ thigh/ knee	Torsion spring	Manual; Stepless locking	2 kg	Muscle activity reduced by 44.8–71.5%, plantar pressure by 58.5–64.2%	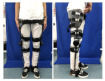	/	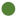 ED
Chang’an University, China [56]	Thigh/ knee	Micro silicone oil liquid spring	/	1.223 kg	Femorotibial joint force reduced by 24.5%, patellofemoral force by 23.8%, quadriceps-ligament force by 21.2%	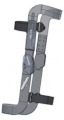	/	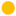 EC (silicone Oil-Based Hydraulic Spring System)
Nagoya University, Japan [57]	Thigh/ knee	Gas spring	Fixed multistage locking	3.7 kg	Physical load on biceps femoris and gluteus maximus was significantly reduced	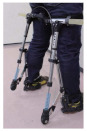	/	/
Harbin Institute of Technology, China [58]	knee	Electric machine	/	1.7 kg	Maximum postural deviation reduced by 49.3% during walking and 71.9% during squatting	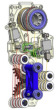	/	/

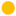
EC—Excessive Cos, 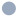
TC—Technological Complexity, 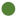
ED—Ergonomic Deficiencies.

**Table 5:** Comparison of lower-limb squat-assistant wearable device types.

Comparison of Lower-Limb Squat-Assistant Wearable Device Types				
	Side-Support	Rear-Support	Medial-Support	Certain Joint Matching
**Main Advantages**	Focuses on lateral leg stability; simple structure.	Rearward center of gravity provides stable support and good energy transfer path.	Good stability, conforms to the leg; high support strength; matches the squatting center of gravity trajectory.	Natural motion, high alignment, closer to biomechanical movement patterns.
**User Experience**	Moderate wearing comfort; lateral supports may restrict some movements.	Stable squatting assistance, but slightly heavy to wear.	Helps maintain lower-limb symmetry and fits closely to the leg, but more sensitive to different body shapes.	Provides greater range of motion and higher comfort and naturalness; sensitive to individual differences.
**Application Scenarios**	Static factory work; frequent sit–stand transitions.	Low-speed walking and moderate activity levels; suitable for long-duration support or rear stability needs.	Long-duration static squatting tasks such as maintenance and assembly.	High-dynamic tasks, such as assisted walking or rehabilitation training.
**Limitations**	Poor stability, high motion interference; difficult to maintain dynamic balance.	Protrudes at the back, limited in confined spaces; significant motion interference; lacks dynamic adaptability.	Not suitable for high-dynamic environments; high body size matching requirements.	Complex structure, high manufacturing difficulty; strong dependence on control systems.
**Usability**	High; quick to use and suitable for beginners.	Moderate; requires adaptation to weight.	Moderate; requires individualized adjustment.	Low to moderate; highly dependent on control, requires training and adaptation.
**Cost**	Low; simple materials and manufacturing.	Medium; slightly more complex structure.	Medium to high; requires custom-fit structure.	High; involves multi-joint structure and control systems.
**Technical Challenges**	Poor dynamic stability; likely to disrupt natural gait.	Weight control and center-of-gravity coordination.	Balancing between size customization and comfort.	High-precision control and sensor fusion; difficult to achieve coordination with the human body.
**Commercialization**	Several lightweight products already on the market; suitable for workers.	High market acceptance; most common in commercial lower-limb assistive devices.	Internal support has entered the commercial market, but much research remains at the laboratory stage.	Mostly in lab validation or pilot application stages; low commercialization level but promising prospects.

**Table 6:** Comfort design objectives and measures.

Design Objective	Specific Measures
Size Adjustability	Implement multi-level or stepless height adjustment [28]
	Adopt modular design with adjustable rod length [34]
	Utilize a three-support rod structure with adjustable height and size [35]
	Integrate adaptive seat board [39]
	Apply three-bar linkage mechanism to accommodate various sitting postures [40]
	Use stepless pneumatic rod adjustment with memory function [47]
	Employ elastic straps and adjustable support frame [54]
Support Stability	Form a triangular support structure upon locking to distribute body weight and reduce lower-limb load [28]
	Integrate triangular support frame [29]
	Use multi-bar and four-bar auxiliary mechanisms to enhance structural stability [38]
	Form triangular support in the seated position to effectively distribute pressure [47]
Pressure Distribution	Fix the seat using an abdominal strap to avoid direct contact with thighs and calves, reducing constraint [19]
	Employ cross-hip or underarm support to transmit force through the hip strap and relieve thigh/calf restraint [22]
	Integrate a load transfer module to shift folded device weight from shoulders to body core [31]
	Apply semi-wearable thigh plates to distribute contact pressure [36]
	Use carbon fiber ankle joint to reduce distal pressure [55]
	Optimize knee joint topology with flexible design to reduce joint pressure [47]
Reduction of Motion Interference	Design footwear allowing foot rotation and tilting (e.g., heel lift) [19]
	Utilize a biased crank–slider mechanism to improve hip flexion range during squatting [20]
	Apply dual support rods aligned with center-of-gravity trajectory to reduce hip constraints [57]
	Use aluminum linkage hinges (0–100° motion) to prevent knee joint torque imbalance [41]
	Implement a six-bar mechanism for dynamic support at various workstation angles [42]
	Employ flexible drive cables to minimize movement restriction [56]
Others	Equip ankle connection with anti-slip rubber or magnetic fasteners to prevent detachment during walking [29]
	Add cushioning pad to the seat for enhanced seated comfort [48]

**Table 7:** Comparative analysis of actuation methods.

Comparative Analysis of Actuation Methods						
Actuation Method	Performance	Volume/Weight	Safety and Stability	Application Scenarios	Limitations	Cost and Commercialization Level
Servo motors [21,22,35,39,45,48,54,58]	Precise control over output timing, amplitude, and motion profile Suitable for programmable movement assistance	Relatively heavy Requires battery power	Sensors and motors sensitive to temperature/humidityRequires regular maintenance	Complex movement assistance Applications demanding fine-grained control	High cost of advanced motors and sensors Increased system complexity	High technological maturity Widely commercialized in wearable robotics
hydraulic drives [20]	High force and torque outputExcellent steady-state performance	Large and bulky	Risk of leakage and pressure instability High maintenance demand	Heavy-duty and high-torque tasks Industrial settings requiring deep squatting or strong resistance	Not suited for daily wearable use High R&D and upkeep costs	Commercialized in industrial-grade exoskeletons
pneumatic cylinder drives [19,31,36]	High instantaneous torque output Durable components with low maintenance	Lightweight system Requires external air supply	Strong interference resistance Stable across uneven terrain	Repetitive sit–stand transitions Static squatting Semi-mobile industrial workstations	Requires air source (tank/compressor)Slow response time Poor portability	Commercialized Low component cost and short development cycle
Pneumatic spring [35,38,57]	Instant reactive counterforce Compact and safe	Compact and lightweight	Environmentally robust High safety in confined spaces	Limited mobility range environmentsBackup support in hybrid systems	Limited output force Slight response delay	Low R&D and maintenance costsCommercial applications already in place
Mechanical Spring [47,49]	Fixed output forceShort response time	Compact and stable	High mechanical stability No electronics involved	Cost-sensitive or lightweight applications Passive or semi-active assistive devices	Cannot adaptively regulate assistance Potential long-term wearability concerns	Low development cost Early-stage commercialization achieved

## References

[1] Kuorinka L., Jonsson B., Kilbom A., Vinterberg H., Biering-Sørensen F., Andersson G., Jørgensen K. (1987). Standardised Nordic questionnaires for theanalysis of musculoskeletal symptoms1. Appl. Ergon..

[2] James S.L., Abate D., Abate K.H., Abay S.M., Abbafati C., Abbasi N., Abbastabar H., Abd-Allah F., Abdela J., Abdelalim A. (2018). Global, regional, and national incidence. prevalence, and years lived with disability for 354 diseases and injuries for 195 countriesand territories, 1990–2017: A systematic analysis for the Global Burden of Disease Study 2017. Lancet.

[3] Zorzenon R., Lizarelli F.L., Daniel B.D. (2022). What is the potential impact of industry 4.0 on health and safety at work?. Saf. Sci..

[4] Lind C.M., Abtahi F., Forsman M. (2023). Wearable motion capture devices for the prevention of work-related musculoskeletal disorders in ergonomics—An overview of current applications, challenges, and future opportunities. Sensors.

[5] Patel V., Chesmore A., Legner C.M., Pandey S. (2022). Trends in workplace wearable technologies and connected-worker solutions for next-generation occupational safety, health, and productivity. Adv. Intell. Syst..

[6] Kawamoto H., Sankai Y. (2002). Power assist system HAL-3 for gait disorder person. Proceedings of the International Conference on Computers for Handicapped Persons.

[7] Santoso G., Sugiharto S., Mughni A., Ammarullah M.I., Bayuseno A.P., Jamari J. (2022). Chairless Chairs for Orthopedic Surgery Purpose–A Literature Review. Open Access Maced. J. Med. Sci..

[8] Abdul Rahman A., Amin Y., Adon M.Y. (2014). Association between awkward posture and musculoskeletal disorders (MSD) among assembly line workers in an automotive industry. Malays. J. Med. Health Sci..

[9] Wijegunawardana I., Ranaweera R.K., Gopura R.A. (2023). Lower extremity posture assistive wearable devices: A review. IEEE Trans. Hum.-Mach. Syst..

[10] Kuber P.M., Alemi M.M., Rashedi E. (2023). A systematic review on lower-limb industrial exoskeletons: Evaluation methods, evidence, and future directions. Ann. Biomed. Eng..

[11] Ma Z., Liu J., Ma G., Gao J., Chen B., Zuo S. (2023). Lockable Lower-Limb Exoskeleton Based on a Novel Variable-Stiffness Joint: Reducing Physical Fatigue at Squatting. J. Mech. Robot..

[12] Li B., Yuan B., Tang S., Mao Y., Zhang D., Huang C., Tan B. (2018). Biomechanical design analysis and experiments evaluation of a passive knee-assisting exoskeleton for weight-climbing. Ind. Robot. Int. J..

[13] Bonner D.R. (1979). Wearable Chair. U.S. Patent.

[14] Tang X., Wang X., Xue Y., Wei P. (2023). An Unpowered Knee Exoskeleton for Walking Assistance and Energy Capture. Micromachines.

[15] Kim J.H., Shim M., Ahn D.H., Son B.J., Kim S.Y., Kim D.Y., Baek Y.S., Cho B.K. (2015). Design of a knee exoskeleton using foot pressure and knee torque sensors. Int. J. Adv. Robot. Syst..

[16] (2019). legX/suitX. https://www.suitx.com/legx.

[17] Pillai M.V., Van Engelhoven L., Kazerooni H. (2020). Evaluation of a lower leg support exoskeleton on floor and below hip height panel work. Hum. Factors.

[18] Wang Z., Wu X., Zhang Y., Chen C., Liu S., Liu Y., Peng A., Ma Y. (2021). A semi-active exoskeleton based on EMGs reduces muscle fatigue when squatting. Front. Neurorobotics.

[19] Mitsuda T., Wakabayashi M., Kawamura S. (2004). Development of wearable chair using pneumatic passive elements. J. Robot. Mechatron..

[20] Wang Z., Wang B., Xu D. (2016). Design and simulation of a lower-limb power-assist exoskeleton for hip joint based on deep squat. Proceedings of the IEEE International Conference on Information and Automation (ICIA).

[21] Sado F., Yap H.J., Ghazilla R.A.R., Ahmad N. (2019). Design and control of a wearable lower-body exoskeleton for squatting and walking assistance in manual handling works. Mechatronics.

[22] Ju H., Li H., Guo S., Fu Y., Zhang Q., Zheng T., Zhao J., Zhu Y. (2024). J-Exo: An exoskeleton with telescoping linear actuators to help older people climb stairs and squat. Sens. Actuators A Phys..

[23] Daines K., Lemaire E.D., Smith A., Herbert-Copley A. (2017). Sit-to-stand and stand-to-sit crutch use for lower extremity powered exoskeletons. Proceedings of the 2017 IEEE International Symposium on Robotics and Intelligent Sensors (IRIS).

[24] Olson J.S. (2021). Design and Development of Exoskeletons for Squatting, Gait Assistance, and Fall Prevention Applications. Ph.D. Thesis.

[25] Bessler-Etten J., Schaake L., Prange-Lasonder G.B., Buurke J.H. (2022). Assessing effects of exoskeleton misalignment on knee joint load during swing using an instrumented leg simulator. J. Neuroeng. Rehabil..

[26] Zanotto D., Akiyama Y., Stegall P., Agrawal S.K. (2015). Knee joint misalignment in exoskeletons for the lower extremities: Effects on user’s gait. IEEE Trans. Robot..

[27] Jiang J., Chen P., Peng J., Qiao X., Zhu F., Zhong J. (2023). Design and optimization of lower limb rehabilitation exoskeleton with a multiaxial knee joint. Biomimetics.

[28] Chairless Chair 2.0. https://www.noonee.com/en/.

[29] Bae K.H., Jung K.M., Kim K.J., Yoon J.Y., Hyun D.J. (2021). Wearable Chair Having Four-Link Structure. U.S. Patent.

[30] Kong Y.K., Park C.W., Cho M.U., Kim S.-Y., Kim M.-J., Hyun D.J., Bae K., Choi J.K., Ko S.M., Choi K.-H. (2021). Guidelines for working heights of the lower-limb exoskeleton (CEX) based on ergonomic evaluations. Int. J. Environ. Res. Public Health.

[31] https://newatlas.com/lex-folding-wearable-chair/56211/.

[32] ExoChair. https://orlovbel.wixsite.com/exochair/main.

[33] “OFREES Chair”. https://www.amazon.com/ofrees-wearable-portable-chairless-155cm-163cm/dp/b07s9wk8lj.

[34] Bijalwan A., Misra A. (2016). Design and structural analysis of flexible wearable chair using finite element method. Open J. Appl. Sci..

[35] Han B., Du Z., Huang T., Zhang T., Li Z., Bai O., Chen X. (2019). Mechanical framework design with experimental verification of a wearable exoskeleton chair. Proceedings of the 2019 International Conference on Robotics and Automation (ICRA).

[36] Zheng H., Shen T., Afsar M.R., Kang I., Young A.J., Shen X. (2019). A semi-wearable robotic device for sit-to-stand assistance. Proceedings of the 2019 IEEE 16th International Conference on Rehabilitation Robotics (ICORR).

[37] Irawan A.P., Utama D.W., Affandi E., Suteja H. (2019). Product design of chairless chair based on local components to provide support for active workers. IOP Conf. Ser. Mater. Sci. Eng..

[38] Han Y., Liu Y., Zhang W. (2021). Design of a passive exoskeleton chair with an auxiliary support mechanism for assembly tasks. Proceedings of the 2021 IEEE International Conference on Robotics and Biomimetics (ROBIO).

[39] Liao Y., Wang C., Wu X., Lu F., Wang P., Cai S. (2015). On the mechanical design and control of a self-adaptive exoskeleton chair. Proceedings of the 2015 IEEE International Conference on Information and Automation.

[40] Wijegunawardana I.D., Kumara M.B.K., De Silva H.H.M.J., Viduranga P.K.P., Ranaweera R.K.P.S., Gopura R.A.R.C., Madusanka D.G.K. (2019). ChairX: A robotic exoskeleton chair for industrial workers. Proceedings of the 2019 IEEE 16th International Conference on Rehabilitation Robotics (ICORR).

[41] Tu Y., Zhu A., Song J., Zhang X., Cao G. (2022). Design and experimental evaluation of a lower-limb exoskeleton for assisting workers with motorized tuning of squat heights. IEEE Trans. Neural Syst. Rehabil. Engineering.

[42] Chenchen L., Zilin X., Fengjie C., Yingchao Z., Yijun Z., Ning D., Xi W. (2022). Optimization design and manufacture of powerless human-assisted exoskeleton chair. Mach. Manuf. Autom..

[43] Huimei K., Jing G., Qian W., Yuqi F., Zehao X., Yishe X. (2023). Unpowered wearable exoskeleton seat structure design and optimization. Equip. Manuf. Technol..

[44] Junius K., Degelaen M., Lefeber N., Swinnen E., Vanderborght B., Lefeber D. (2017). Bilateral, Misalignment-Compensating, Full-DOF Hip Exoskeleton: Design and Kinematic Validation. Appl. Bionics Biomech..

[45] Ikeuchi Y., Ashihara J., Hiki Y., Kudoh H., Noda T. (2009). Walking assist device with bodyweight support system. Proceedings of the 2009 IEEE/RSJ International Conference on Intelligent Robots and Systems.

[46] Al-Shuka H.F.N., Rahman M.H., Leonhardt S., Ciobanu I., Berteanu M. (2019). Biomechanics, actuation, and multi-level control strategies of power-augmentation lower extremity exoskeletons: An overview. Int. J. Dyn. Control..

[47] Lee K.M., Wang D. (2015). Design analysis of a passive weight-support lower-extremity-exoskeleton with compliant knee-joint. Proceedings of the 2015 IEEE International Conference on Robotics and Automation (ICRA).

[48] Wulong H., Xin’an W., Zheng X. (2016). A Weight-Supporting Wearable Robot for Walking Assist. Proceedings of the 7th International Conference on Intelligent Human-Machine Systems and Cybernetics.

[49] Lovrenovic Z., Doumit M. (2019). Development and testing of a passive walking assist exoskeleton. Biocybern. Biomed. Eng..

[50] Bhardwaj S., Khan A.A., Muzammil M. (2021). Lower limb rehabilitation robotics: The current understanding and technology. Work.

[51] Li J., Zhang Z., Tao C., Ji R. (2015). Structure design of lower limb exoskeletons for gait training. Chin. J. Mech. Eng..

[52] Archelis. https://www.archelis.com/.

[53] Jiashuo C. (2019). Design and Analysis of Lower Extremity Exoskeleton for Gravity Support During Squatting. Ph.D. Thesis.

[54] Chen S., Stevenson D.T., Yu S., Mioskowska M., Yi J., Su H., Trkov M. (2021). Wearable es for kneeling tasks in construction. IEEE/ASME Trans. Mechatron..

[55] Yan Z., Han B., Du Z., Huang T., Bai O., Peng A. (2021). Development and testing of a wearable passive lower-limb support exoskeleton to support industrial workers. Biocybern. Biomed. Eng..

[56] Xuan Z., Shuo F., Zhenxian C., Jing Z., Zhongmin J. (2022). Design idea and biomechanical analysis of a liquid spring self-force source knee assist orthosis. J. Biomed. Eng..

[57] Hasegawa Y., Hoshino T., Tsukahara A. (2016). Wearable assistive device for physical load reduction of caregiver-adaptive to caregiver’s motion during transferring support. Proceedings of the 2016 World Automation Congress (WAC).

[58] Yu S., Huang T.H., Di Lallo A., Zhang S., Wang T., Fu Q., Su H. (2022). Bio-inspired design of a self-aligning, lightweight, and highly-compliant cable-driven knee exoskeleton. Front. Hum. Neurosci..

[59] Lu T.W., Chang C.F. (2012). Biomechanics of human movement and its clinical applications. Kaohsiung J. Med. Sci..

[60] Kadaba M.P., Ramakrishnan H.K., Wootten M.E. (1990). Measurement of lower extremity kinematics during level walking. J. Orthop. Res..

[61] Yumin H., Ting L., Siyu G., Jing Z., Yang L., Weiqi L. (2023). A review of kinematics and dynamics of lower extremity exoskeleton. J. Ordnance Equip. Eng..

[62] Tarokh M., Lee M. (2008). Kinematics modeling of multi-leggedrobots walking on rough terrain. Proceedings of the Second International Conference on Future Generation Communication and Networking Symposia.

[63] Mohdzawawi M.Z.F., Elamvazuthi I., Aziz A.A., Mazlan S.F., Ku Abd Rahim K.N. (2016). Dynamie analysis of three degree of freedom (3-D0F) exoskeleton for lower extremities. Proceedings of the 2016 2nd EEE International Symposium on Robotics and Manufacturing Automation (ROMA).

[64] Sun H., Zhang L., Li C. (2009). Dynamic analysis of horizontal lower limbs rehabilitativerobot. Proceedings of the IEEE International Conference on Intelligent Computing and Intelligent Systems IEEE.

[65] Yajing W. (2020). Biomechanical Study of Human Knee Joint Based on Dynamic Capture and Foot Pressure Test. Ph.D. Thesis.

[66] Xiao L., Jianjun Z., Kaicheng Q., Gaowei Y. (2020). Lower extremity exoskeleton variable axis knee joint configuration design and rod length optimization. Mech. Sci. Technol..

[67] Wei S., Anmin X., Yubao Z. (2008). Modeling and Simulation of Robot Dynamics Based on Kane ’s Method. Microcomput. Inf..

[68] Junxia Z., Youzhi Y.I., Quan W. (2015). Structuredesign and simulation of dynamic walking-aid. J. Mach. Des..

[69] Gang T. (2011). Biomechanics Simulation Analysis for Typical Movements of Human. Ph.D. Thesis.

[70] Qi Q., Cheng S., Zhang D., Zhou M., Yu H. (2017). Mechanism design, kinematics and dynamics analysis of wearable seats. Tech Wind..

[71] Zhanteng G. (2020). Human Squat Motion Analysis and Exoskeleton Power Control Strategy Research. Ph.D. Thesis.

[72] Jianhui W., Xiulin X. (2012). Human lower limb dynamics modeling and simulation research status. Rehabil. Theory Pract. China.

[73] Murphy M.C. (1990). Geometry and the Kinematics of the Normal Human Knee. Ph.D. Thesis.

[74] Zlotnicki J.P., Naendrup J.H., Ferrer G.A., Debski R.E. (2016). Basic biomechanic principles of knee instability. Curr. Rev. Musculoskelet. Med..

[75] Celebi B., Yalcin M., Patoglu V. (2013). AssistOn-Knee: A self-aligning knee exoskeleton. Proceedings of the 2013 IEEE/RSJ International Conference on Intelligent Robots and Systems.

[76] Bing C., Minzhou L., Shaoming S., Meiling W., Kun W. (2014). Design of knee joint of energy-saving and vibration-reducing humanoid robot based on bionic principle. Robot.

[77] Zhu A., Shen Z., Shen H., Wu H., Zhang X. (2018). Design of a passive weight-support exoskeleton of human-machine multi-link. Proceedings of the 2018 15th International Conference on Ubiquitous Robots (UR).

[78] Jiyuan S., Aibin Z., Yao T., Xinyu W., Yulin Z., Xu Z. (2021). Prediction of the expected angle of lower limb exoskeleton joints by human-computer interaction force. J. Xi ’an Jiaotong Univ..

[79] Luo S., Meng Q., Li S., Yu H. (2024). Research of intent recognition in rehabilitation robots: A systematic review. Disabil. Rehabil.: Assist. Technol..

[80] Wenyuan L., Sheng B. (2019). Perceptual interaction and control strategy of active rehabilitation training robot. Technol. News.

[81] Rechy-Ramirez E.J., Hu H. (2015). Bio-signal based control in assistive robots: A survey. Digit. Commun. Netw..

[82] Reaz M.B.I., Hussain M.S., Mohd-Yasin F. (2006). Techniques of EMG signal analysis: Detection, processing, classification and applications. Biol. Proced. Online.

[83] Inoue T., Matsuo R. (2020). Prediction of Sit-to-Stand Time Using Trunk Angle and Lower Limb EMG for Assistance System. Proceedings of the 2020 IEEE 2nd International Conference on Artificial Intelligence in Engineering and Technology (IICAIET).

[84] Wenjie Z., Xinqin J., Xiaodong W., Teaching Guidance Subcommittee of Industrial Design Specialty in Colleges and Universities of the Ministry of Education, Industrial Design Branch of China Mechanical Engineering Society, Industrial Design Teaching Committee of China Mechanical Industry Education Association Research on squatting operation based on surface electromyography. Proceedings of the 2016 National Industrial Design Education Seminar and International Industrial Design Summit Forum.

[85] Yonglin H., Qing T., Xiaodong Z., Qingzheng C. (2023). Electromyographic prediction method for multi-joint continuous motion of human lower limbs. Mach. Hydraul..

[86] Lloyd D.G., Besier T.F. (2003). An EMG-driven musculoskeletal model to estimate muscle forces and knee joint moments in vivo. J. Biomech..

[87] Xiaodong Z., Jiangcheng C., Gui Y. (2018). EMG sensing and human-computer interaction control method of lower limb rehabilitation robot. Vibration.Test Diagn..

[88] Wenfeng L., Zhigang Y., Xinyun H. (2019). Effect of EMG signal selection on continuous motion estimation of lower limb joints. Mech. Des. Manuf..

[89] Mohebbi A. (2020). Human-robot interaction in rehabilitation and assistance: A review. Curr. Robot. Rep..

[90] Cao J., Xie S.Q., Das R., Zhu G.L. (2014). Control strategies for effective robot assisted gait rehabilitation: The state of art and future prospects. Med. Eng. Phys..

[91] Meng W., Liu Q., Zhou Z., Ai Q., Sheng B., Xie S. (2015). Recent development of mechanisms and control strategies for robot-assisted lower limb rehabilitation. Mechatronics.

[92] Shouyin L., Luhao Y. (2021). Research progress of human-computer interaction control technology for rehabilitation robots. J. Shandong Jianzhu Univ..

[93] Unluhisarcikli O., Pietrusinski M., Weinberg B., Bonato P., Mavroidis C. Design and control of a robotic lower extremity exoskeleton for gait rehabilitation. Proceedings of the Intelligent Robots and Systems (IROS) 2011.

[94] Wei W., Shijia Z., Yuxuan X., Jihua G., Xichuan L. (2020). A hip active assisted exoskeleton that assists the semi-squat lifting. Appl. Sci..

[95] Ke W., Xin T. (2022). A review of human-computer interaction control based on exoskeleton robots. Mech. Eng..

[96] Yuqing X. (2020). Review on human-computer interaction design of walking-aid rehabilitation robot. Pack. Eng..

[97] EKSOBIONICS Ekso GT Robotic Exoskeleton Cleared by FDA for Use with Stroke and Spinal Cord Injury Patients. http://ir.eksobionics.com/press-releases/detail/570/ekso-gt-roboticexoskeletoncleared-by-fda-for-use-with.

[98] Wu Z., Yang M., Xia Y., Wang L. (2023). Mechanical structural design and actuation technologies of powered knee exoskeletons: A review. Appl. Sci..

[99] Tang X., Wang X., Xue Y., Yin R., Yang J. (2023). A study of knee exoskeleton configuration based on lower limb motion characteristics analysis. Machines.

[100] Sigmund O., Maute K. (2013). Topology optimization approaches: A comparative review. Struct. Multidiscip. Optim..

[101] Winter D.A. (2009). Biomechanics and Motor Control of Human Movement.

[102] Malcolm P., Galle S., Derave W., De Clercq D. (2018). Bi-articular knee-ankle-foot exoskeleton produces higher metabolic cost reduction than weight-matched mono-articular exoskeleton. Front. Neurosci..

[103] Li Y., Guan X., Han X., Tang Z., Meng K., Shi Z., Penzlin B., Yang Y., Ren J., Yang Z. (2020). Design and preliminary validation of a lower limb exoskeleton with compact and modular actuation. IEEE Access.

[104] Tiboni M., Borboni A., Vérité F., Bregoli C., Amici C. (2022). Sensors and actuation technologies in exoskeletons: A review. Sensors.

[105] Li Y., Gan J. (2022). Multidisciplinary evaluation metrics for the usability of wearable chairs. Proceedings of the 2022 International Congress on Human-Computer Interaction, Optimization and Robotic Applications (HORA).

[106] Yang K., Jiang Q.F., Wang X.L., Chen Y.W. (2018). Structural design and modal analysis of exoskeleton robot for rehabilitation of lower limb. Journal of Physics: Conference Series.

[107] Li Y., Wang X., Xu P., Zheng D., Liu W., Wang Y., Qiao H. (2013). SolidWorks/SimMechanics-based lower extremity exoskeleton modeling procedure for rehabilitation. Proceedings of the World Congress on Medical Physics and Biomedical Engineering.

[108] Lei T. (2020). Research on Motion Reliability Analysis Method of Lower Extremity Exoskeleton Mechanism. Ph.D. Thesis.

[109] Songcheng X., Rongsong Y., Qinghua Z. (2021). Random vibration analysis of RV reducer based on ANSYS Workbench. Mech. Transm..

[110] Li H., Sui D., Ju H., An Y., Zhao J., Zhu Y. (2022). Mechanical compliance and dynamic load isolation design of lower limbexoskeleton for locomotion assistance. IEEE/ASME Trans. Mechatron..

[111] Buldt A.K., Allan J.J., Landorf K.B., Menz H.B. (2018). The relationship between foot posture and plantar pressure during walking in adults: A systematic review. Gait Posture.

[112] Moudy S.C., Tillin N.A., Sibley A.R., Strike S. (2020). Foot strike alters ground reaction force and knee load when stepping down during ongoing walking. Gait Posture.

[113] Abe D., Fukuoka Y., Muraki S., Yasukouchi A., Sakaguchi Y., Niihata S. (2011). Effects of load and gradient on energy cost of running. J. Physiol. Anthropol..

[114] Luger T., Seibt R., Cobb T.J., Rieger M.A., Steinhilber B. (2019). Influence of a passive lower-limb exoskeleton during simulated industrial work tasks on physical load, upper body posture, postural control and discomfort. Appl. Ergon..

[115] Gams A., Petrič T., Debevec T., Babič J. (2013). Effects of robotic knee exoskeleton on human energy expenditure. IEEE Trans. Biomed. Eng..

[116] Wioland L., Atain Kouadio J.J., Bréard H., Clerc-Urmès I., Paty B. (2025). The Adoption of Occupational Exoskeletons: From Acceptability to Situated Acceptance, Questionnaire Surveys. Int. J. Hum. Comput. Interact..

[117] Mohammed El Husaini M., Maberry A., Martin A.E. (2023). Validation of a modified visual analogue scale to measure user-perceived comfort of a lower-limb exoskeleton. Sci. Rep..

[118] Chae U.R., Kim K., Choi J., Hyun D.J., Yun J., Lee G.H., Hyun Y.G., Lee J., Chung M. (2021). Systematic usability evaluation on two harnesses for a wearable chairless exoskeleton. Int. J. Ind. Ergon..

[119] Hoffmann N., Prokop G., Weidner R. (2022). Methodologies for evaluating exoskeletons with industrial applications. Ergonomics.

[120] Cha J.S., Monfared S., Stefanidis D., Nussbaum M.A., Yu D. (2020). Supporting surgical teams: Identifying needs and barriers for exoskeleton implementation in the operating room. Hum. Factors.

[121] Gensheng L., Guoning Y., Fei X. (2018). Research progress of lower extremity exoskeleton robot control strategy. Chin. J. Rehabil. Med..

[122] Kwok T.H., Wang C.C. (2014). Shape optimization for human-centric products with standardized components. Comput.-Aided Des..

[123] Chester M.R., Rys M.J., Konz S.A. (2002). Leg swelling, comfort and fatigue when sitting, standing, and sit/standing. Int. J. Ind. Ergon..

[124] (2014). Robots and Robotic Devices—Safety Requirements for Personal Care Robots.

